# Associations of Socioeconomic Status and Physical Activity With Obesity Measures in Rural Chinese Adults

**DOI:** 10.3389/fpubh.2020.594874

**Published:** 2021-01-08

**Authors:** Mingming Pan, Runqi Tu, Jianjun Gu, Ruiying Li, Xiaotian Liu, Ruoling Chen, Songcheng Yu, Xian Wang, Zhenxing Mao, Wenqian Huo, Jian Hou, Chongjian Wang

**Affiliations:** ^1^Department of Epidemiology and Biostatistics, College of Public Health, Zhengzhou University, Zhengzhou, China; ^2^Department of Neurosurgery, Zhengzhou University People's Hospital, Henan Provincial People's Hospital, Zhengzhou, China; ^3^Faculty of Education, Health and Well-being, University of Wolverhampton, Wolverhampton, United Kingdom

**Keywords:** physical activity (exercise), obesity, rural population, gender difference, socioeconomic status

## Abstract

**Background:** Although independent association of socioeconomic status (SES) or physical activity (PA) with obesity has been well-documented in urban settings, their independent and joint associations on obesity measures are limited in rural regions.

**Methods:** Almost 38,000 (*n* = 37,922) individuals were included from the Henan Rural Cohort Study. The International Physical Activity Questionnaire (IPAQ) was used to evaluate PA. Obesity was reflected by body mass index (BMI), waist circumference (WC), waist-to-hip ratio (WHR), waist-to-height ratio (WHtR), body fat percentage (BFP), and visceral fat index (VFI). The independent and interactive effects of SES and PA on obesity were analyzed by logistic regression models and generalized linear regression models, respectively.

**Results:** Compared with high education level, the OR (95%CI) of obesity defined by BMI with low education level was 1.466 (1.337, 1.608), 1.064 (0.924, 1.225), and 1.853 (1.625, 2.114) in total population, men and women, respectively. Besides, the OR (95%CI) of obesity defined by BMI associated with per capita monthly income were 1.089 (1.015, 1.170), 1.192 (1.055, 1.347), 1.038 (0.951, 1.133) in total population, men and women, respectively. Similar results had been observed in other obesity measures. Negative interactive association of low education level and PA on obesity measures were observed only in women (all *P* < 0.05).

**Conclusions:** This study suggests that women are more susceptible to obesity concerning low SES and that adequate PA may be a potential target for mitigating the negative effect of low SES on obesity in women.

**Clinical Trial Registration:** The Henan Rural Cohort Study has been registered at Chinese Clinical Trial Register (Registration number: ChiCTR-OOC-15006699) http://www.chictr.org.cn/showproj.aspx?proj=11375.

## Introduction

Obesity is a growing and disturbing global public health crisis (1). According to the Global Burden of Disease statistics in 2017, more than four million people died each year as a result of being overweight or obese (2). It turns out that excess weight is the main risk factor for various diseases, especially stroke and coronary heart disease (3–5). Body mass index (BMI) has been widely used for defining obesity. However, BMI alone does not fully characterize adiposity, and other anthropometric measures have been proposed to define obesity, such as waist circumference (WC) (6), waist-to-hip ratio (WHR) (7), waist-to-height ratio (WHtR) (8), body fat percentage (BFP) (9), and visceral fat index (VFI) (10), etc. They define obesity according to different anthropometric emphasis with different predictive capabilities for diseases. Besides, the prevalence of obesity may vary greatly with different measures used to define obesity. However, it is still controversial which measure is most appropriate to define obesity, we therefore used these six objectively measured anthropometric methods to more accurately monitor obesity in the current study.

It is well-known that the socio-economic environment has a significant impact on the prevalence of a high number of diseases, including obesity (11, 12) as it influences people's attitudes, experiences, and access to several health risk factors (13, 14). Based on several systematic reviews (15, 16) of socioeconomic inequalities in obesity, it was found that the relationship between SES and obesity varies across countries with different levels of development. In developed countries, socioeconomically disadvantaged groups tend to have a higher prevalence of obesity, i.e., people with lower income and/or lower levels of education tend to be more likely to be obese. In developing countries, the SES-obesity relationship was found to be more complex: in low-income developing countries, those living in more affluent circumstances are more likely to experience overweight and obesity for both men and women, whereas in middle-income countries, the relationship between SES and obesity is largely mixed for men and predominantly negative for women (15). Notably, gender appears to play an important role in the SES-obesity relationship, and it was said that ignoring gender differences when examining the SES-obesity association may lead to targeting of wrong populations for reducing obesity prevalence and its resultant socioeconomic gradients (17). Additionally, researches on the SES-obesity association were mainly concentrated in developed countries and developing urban cities, with very limited research in rural areas of developing countries. Considering the serious epidemic of obesity and overweight in developing countries, especially in rural settings (18), the SES-obesity associations in these settings were also meriting focus.

It had been well-documented that regular physical activity (PA) has great benefits for keeping fit (19). According to Xiao et al., compared to physically inactive male respondents, physically active ones had about one quarter lower risk of being obese (20). Furthermore, it had been shown that in the SES-obesity relationships, PA could play a significant role (21, 22). For instance, Merino et al. suggested that promoting PA would contribute to preventing obesity for low SES-individuals (21). However, the association between SES and obesity whether affected by PA and how affected by PA was not available in rural regions.

Therefore, the purpose of this study was to investigate the independent and potential joint associations of SES and PA on different obesity measures among participants of different genders from the baseline survey of the Henan Rural Cohort Study.

## Materials and Methods

### Study Population

The Henan Rural Cohort Study was conducted in Henan, China, using a multistage stratified cluster sampling method to recruit a total of 39,259 individuals as a baseline cohort from five rural areas (Suiping, Yuzhou, Xinxiang, Tongxu, and Yima) between 2015 and 2017 (23). Almost 38,000 (*n* = 37,922) individuals aged 18–79 years were included for the further analysis, after excluding individuals with missing data on per capita monthly income, PA, height, weight, waist or hip circumference, body fat percentage (BFP), and visceral fat index (VFI). This study was conducted under the Declaration of Helsinki. Zhengzhou University Life Science Ethics Committee had approved this survey and all participants had signed written informed consent.

### Assessment of Obesity

Anthropometric measures of obesity include waist and hip circumference, height, weight, BFP, and VFI. Weight, BFP, and VFI data were measured using a bioelectrical impendence analysis device (OMRON V. BODY HBF-371) following its operating instructions. Body weight was measured with light clothes to an accuracy of 0.1 kg. Height was measured by taking off shoes and leaning against a calibrated wall. Waist and hip circumferences were measured 1.0 cm above the navel and at the highest hip level, respectively. Height and waist and hip circumference were measured at least twice, each accurately to 0.1 cm, with the difference between the two measurements <0.5 cm and averaged for statistical analysis. The details of the anthropometric measures have been described elsewhere (18).

BMI is calculated as weight (kg) divided by height squared (m^2^), WHR is calculated as WC (cm) divided by hip circumference (cm), and WHtR is calculated as WC (cm) divided by height (cm). Individuals with a BMI ≥ 28 kg/m^2^ were defined as obese individuals, following the Working Group on Obesity in China (24). The cut-off values for the other five obesity measures were set based on previous criterion as follows: WC, men/women ≥ 90/80cm (6); WHR, men/women ≥ 0.90/0.85 (7); WHtR ≥ 0.5 (8); BFP, men/women ≥ 25%/30% (9); VFI ≥ 10 (25).

### Assessment of SES

Education level and per capita monthly income were used as proxies of SES, which were consistent with previous studies (20, 26). Education level was derived from the item “educational level” in the questionnaire. Options include illiteracy, primary school, junior high school, senior high school/technical secondary school, university/ junior college, and postgraduate. They were finally divided into three groups: low (illiteracy or primary school), medium (junior high school), and high (senior high school or above). Per capita monthly income was a continuous indicator obtained by dividing the average annual income by the number of household members. Due to the discrete nature of the data, a logarithmic transformation was used for statistical analysis.

### Assessment of PA

The levels of PA were assessed by the International Physical Activity Questionnaire (IPAQ) (27, 28). Participants were asked about the amount of time spent on vigorous activity, moderate activity, and walking over the past week, and the total metabolic equivalent (MET) value was estimated by combining the different types of PA during the week with the corresponding coefficients. The detailed procedure had been described in previous studies (29). Briefly, 1 MET was defined as the amount of energy expended by an individual while sitting quietly. The coefficients of compendium average MET were eight for vigorous activity; four for moderate activity; and 3.3 for walking. PA was classified into three levels: low, moderate, and high. Classification of PA as high shall meet one of the following two criteria: (1) Vigorous activity at least 3 days/week, Mets at least 1,500 MET-min/week. (2) Any combination of the three exercise types (vigorous activity, moderate activity, or walking) for at least 5 days/week and accumulating Mets of at least 3,000 MET-min/week; A classification of PA as moderate shall meet one of the following four criteria: (1) At least 20 min of vigorous activity 3 days a week. (2) Moderate activity for at least 30 min 5 days a week. (3) Walking for at least 30 min per day 5 or more days a week. (4) Accumulated Mets of at least 600 MET-min/week.

### Assessment of Covariates

In addition to SES indicators and PA, we have collected for other variables associated with obesity: region (30), age, gender, marital status, smoking, alcohol consumption, adequate fruit and vegetable intake, and high-fat diet (18, 25). All variables were collected by trained interviewers through face-to-face interviews. Marital status was categorized into married/cohabitation, divorced/widowed/separated and unmarried groups. Smoking and drinking status were categorized into current, former, and never groups. Dietary habits included high (≥500 g/day) and low (<500 g/day) fruit and vegetable intake groups, and high (≥75 g/day) and low-fat diet (<75 g/day) groups (31). The dietary data were collected via a food frequency questionnaire (FFQ) covering the intake of food groups during the previous 12 months. Based on five consumption frequencies (never, day, week, month, year), participants were asked about the amount of food consumed (kg, g). The reliability and validity of FFQ have been conducted and published elsewhere (32). Briefly speaking, the reliability of the FFQ was established by comparing two administrations of the FFQ over a 4-week period while relative validity was established against a 24 h diet recalls (24DR), and the results recommended that our FFQ is appropriate for ranking participants according to food group intake of a rural population.

### Statistical Analysis

Categorical variables and continuous variables were expressed as number (percentage) and median (interquartile range), respectively. The differences in continuous and categorical variables between different genders were analyzed by the Mann-Whitney *U* test and Chi-square test, respectively. We calculated Pearson correlation coefficients among SES indicators, PA and obesity measures, and correlation maps were used to show the direction (negative or positive) and magnitude (strength) of correlation among SES indicators, PA and obesity measures. The independent associations between SES indicators, PA, and obesity measures (dichotomous form) were assessed by using logistic regression models. Interaction associations of SES indicators and PA on obesity measures were conducted by generalized linear regression models and presented by Interaction plots which exhibited how the estimated associations of SES indicators on obesity measures were affected by altered PA intensity. Backward stepwise approaches were used to select covariates for the multivariate analysis, and all explanatory variables with a *P*-value <0.05 were included, including region, age, marital status, smoking status, drinking status, fruit and vegetable intake, high fat diet, education level, per capita monthly income, and PA. Besides, a sensitivity analysis was conducted on the BMI cut-off values of 30 kg/m^2^ (33) to assess the robustness of the main findings. R software version 3.5.1 and SPSS version 21.0 were used for data processing and analysis. All statistical significance was set a *P* < 0.05 at two tails.

## Results

### Basic Characteristics of the Study Population

Table 1 showed the demographic characteristics of the 37,922 participants aged 18–79 years old. The median (interquartile range) age of the total population was 56 (17) years, with men having a higher median (interquartile range) age than women (59 (17) vs. 55 (16), *P* < 0.001). Other selected variables included region, age, education level, smoking status, drinking status, adequate vegetable and fruit intake, high fat diet, PA, as well as obesity measures, which were distributed differently by gender (all *P* < 0.001).

**Table 1:** Characteristics of the study population.

**Variables**	**Overall**	**Men**	**Women**	***P***
	**(*N* = 37,922)**	**(*N* = 14,877)**	**(*N* = 23,045)**	
Region (*n*, %)				<0.001^a^
Yuzhou	8,995 (23.7)	3,132 (21.1)	5,863 (25.4)	
Suiping	15,716 (41.4)	6,477 (43.5)	9,239 (40.1)	
Tongxu	2,464 (6.5)	994 (6.7)	1,470 (6.4)	
Xinxiang	9,796 (25.8)	3,972 (26.7)	5,824 (25.3)	
Yima	951 (2.5)	302 (2.0)	649 (2.8)	
Age [years, median (interquartile range)]	56 (17)	59 (17)	55 (16)	<0.001^b^
Marital status (*n*, %)				0.502^a^
Married/cohabitation	34,103 (89.9)	13,398 (90.1)	20,705 (89.8)	
Unmarried/divorced/widowed	3,819 (10.1)	1,479 (9.9)	2,340 (10.2)	
Education level (*n*, %)				<0.001^a^
Low	16,941 (44.7)	4,992 (33.6)	11,949 (51.9)	
Medium	15,211 (40.1)	6,917 (46.5)	8,294 (36.0)	
High	5,770 (15.2)	2,968 (20.0)	2,802 (12.2)	
Log-transformed per capita monthly income [median (interquartile range)]	3.9 (0.5)	3.9 (0.5)	3.9 (0.5)	0.807^b^
Smoking status (*n*, %)				<0.001^a^
Never	27,652 (72.9)	4,693 (31.5)	22,959 (99.6)	
Ever	3,065 (8.1)	3,044 (20.5)	21 (0.1)	
Current	7,205 (19.)	7,140 (48.)	65 (0.3)	
Drinking status (*n*, %)				<0.001^a^
Never	29,324 (77.3)	6,943 (46.7)	22,381 (97.1)	
Ever	1,743 (4.6)	1,682 (11.3)	61 (0.3)	
Current	6,855 (18.1)	6,252 (42.0)	603 (2.6)	
High fat diet (yes, *n*, %)	7,252 (19.1)	3,731 (25.1)	3,521 (15.3)	<0.001^a^
Adequate vegetable and fruit intake (yes, *n*, %)	21,896 (57.7)	6,455 (43.4)	9,571 (41.5)	<0.001^a^
PA-MET [hour/day, median (interquartile range)]	17.0 (9.9)	17.0 (15.1)	17.5 (9.8)	<0.001^b^
Physical activity (*n*, %)				
Low	11,636 (30.7)	4,998 (33.6)	6,638 (28.8)	<0.001^a^
Moderate	14,652 (38.6)	4,263 (28.7)	10,389 (45.1)	
High	11,634 (30.7)	5,616 (37.7)	6,018 (26.1)	
Obesity (*n*, %)				
BMI	6,693 (17.6)	2,337 (15.7)	4,356 (18.9)	<0.001^a^
WC	19,547 (51.5)	5,152 (34.6)	14,395 (62.5)	<0.001^a^
WHR	23,731 (62.6)	8,226 (55.3)	15,505 (67.3)	<0.001^a^
WHtR	25,809 (68.1)	9,166 (61.6)	16,643 (72.2)	<0.001^a^
BFP	25,672 (67.7)	7,066 (47.5)	18,606 (80.7)	<0.001^a^
VFI	16,751 (44.2)	9,403 (63.2)	7,348 (31.9)	<0.001^a^

*SD, standard deviation; MET, metabolic equivalent; BMI, body mass index; WC, waist circumference; WHR, waist-to-hip ratio; WHtR, waist-to-height ratio; BFP, body fat percentage; VFI, visceral fat index*.

a *Chi-square test was used to test the distributions of categorical variables between genders*.

b *Mann-Whitney U Test was used to compare the difference of continuous variables between genders*.

### Independent Associations Between SES Indicators, PA and Obesity Measures

**Figure 1:**
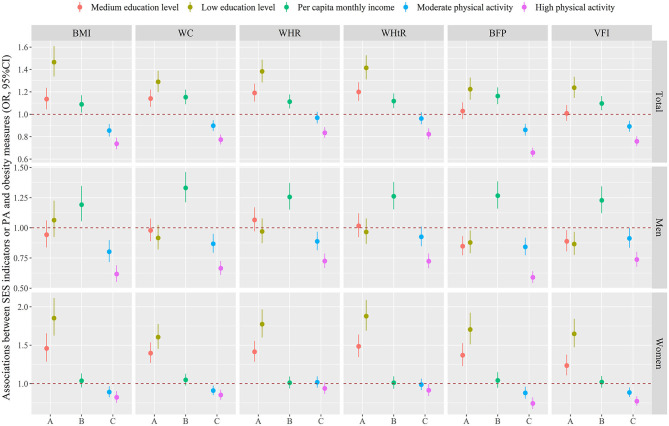
Associations between education level (A) or per capita monthly income (B) or PA (C) and obesity measures. Logistic regression analyses were performed using a fully adjusted model that adjusted for region, age, marital status, smoking status, drinking status, fruit and vegetable intake, high fat diet, SES indicators or PA. The dots and lines were exhibited the estimated regression coefficient and corresponding 95% confidence intervals, respectively. SES, socioeconomic status; PA, physical activity; BMI, body mass index; WC, waist circumference; WHR, waist-to-hip ratio; WHtR, waist-to-height ratio; BFP, body fat percentage; VFI, visceral fat index.

Figure 1 and Supplementary Table 1 showed the independent associations between SES indicators or PA and obesity measures. Logistic regression analyses were performed using a fully adjusted model that adjusted for region, age, marital status, smoking status, drinking status, fruit and vegetable intake, high fat diet, SES indicators or PA. The results showed that the associations of PA with obesity measures were the same both in the total population and across gender, the higher the level of PA, the lower the odds of obesity measures. However, the associations of SES indicators with obesity measures vary across different populations. In terms of education level, compared with high education level, the OR (95%CI) of obesity defined by BMI in total population, men and women with low education level were 1.466 (1.337, 1.608), 1.064 (0.924, 1.225), and 1.853(1.625, 2.114), respectively. Besides, the OR (95%CI) of obesity defined by BMI associated with per capita monthly income were 1.089 (1.015, 1.170), 1.192 (1.055, 1.347), 1.038(0.951, 1.133) in total population, men and women, respectively. Similar results had been observed in other obesity measures. Supplementary Figure 1 displayed the correlation between SES indicators, PA, and obesity measures. PA showed a negative correlation with obesity measures in both the total population and different genders. Education level and per capita monthly income were positively correlated with obesity measures in men, whilst negatively correlated with obesity measures in women.

### Combined Associations of SES Indicators and PA With Obesity Measures

**Figure 2:**
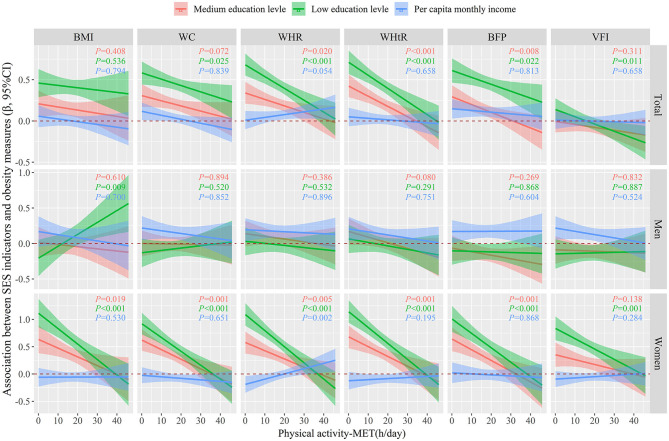
Regression associations between SES indicators and obesity measures (dichotomous) as a function of PA were evaluated by using generalized linear models, after adjusting for age, marital status, smoking status, drinking status, fruit and vegetable intake, high fat diet, education level or per capita monthly income. The lines and areas represented the estimated regression coefficient and 95% confidence intervals, respectively. SES, socioeconomic status; PA, physical activity; BMI, body mass index; WC, waist circumference; WHR, waist-to-hip ratio; WHtR, waist-to-height ratio; BFP, body fat percentage; VFI, visceral fat index.

Figure 2 showed regression associations of SES indicators on obesity measures (dichotomous form) classified by BMI, WC, WHR, WHtR, BFP, or VFI as a function of PA by using generalized linear models in a fully adjusted model, which adjusted for region, age, marital status, smoking status, drinking status, fruit and vegetable intake, high fat diet, education level or per capita monthly income. We did not observe any interaction association between per capita monthly income and PA on obesity measures, neither in the total population nor in different genders. However, there were significant negative interactive associations between low or medium education level and PA on obesity measures both in total population and in women (all *P* < 0.05), which implies that in women, the positive association between low or medium education level and obesity weakens with increasing PA intensity.

### Sensitivity Analysis

The results of the sensitivity analysis were shown in Supplementary Table 2. The estimated associations between SES indicators or PA and obesity measures defined by BMI remained fairly robust after using different cut-off values of BMI.

## Discussion

In this study, the results suggested that: first, a negative association between PA and obesity was observed in both men and women. Second, there were gender differences in the independent and joint associations between SES indicators and obesity measures. The significant inverse association between education level and obesity measures was found only in women, whereas the significant positive association between monthly per capita income and obesity measures was found only in men. Third, we found a negative interaction association between PA and low education level on obesity measures in women.

PA can maintain a healthy weight and reduce the risk of many health problems, including obesity (34–36). A broad range of strategies are recommended to reduce the prevalence of obesity, and PA remains the most common treatment. PA offsets by increased energy consumption and positive energy balance to reduce diet-induced weight gain (37). Additionally, PA has a far-reaching impact on the normal function of the immune and endocrine system (38, 39), as well as the reduction of inflammation and oxidative stress (40, 41), which may help protect individuals from the development of obesity (42, 43). However, according to our previous study in 2019 (29), physical inactivity, and sedentary behavior (sitting for >7.5 h per day) were high in rural areas. The age-standardized prevalence of light PA was 32.74%, higher than the global PA level (44). Besides, the level of sedentary behavior was 26.88%, which was higher compared with a previous study of a 20-country comparison of sitting (45). A major difference between urban and rural environments is that for some rural residents, there may be additional barriers to regular active PA, including isolation, climate, safety fears, cost, lack of transportation, and lack of PA spaces (46). Taking steps to enhance features such as playgrounds, parks, and recreation facilities in rural environments and to reduce barriers to active PA may be good ways to promote active living and address the issue of obesity in rural areas.

It is well-known that SES has an impact on human health status. Extensive research has been carried out to explore the relationship between SES and obesity. However, the results are inconclusive. We found that higher education level was associated with lower odds of obesity in women, which is consistent with some studies in the Philippines (47), Thailand (48), Tehran (49), and urban areas such as Zhejiang (20), Tianjin (26), Guangdong (50), and 33 communities of Northeast China (51). Besides, it was observed in the present study that, men with higher income had higher odds of obesity, which is in agreement with studies in Thailand (48), Mexican (52), and other studies in China (20, 51), but differs from studies in Molise adults (53) and Brazil adults (54). A recent review (16) concluded that in low-income countries, overweight and obesity are more common in more socioeconomically affluent groups and that this pattern flattens and then reverses as country-level income increases. The complex pattern of the SES-obesity relationship highlights the profound influence of social context on obesity, as the social environment can affect people's health in many ways (55), like economic and social development, employment levels, changes in dietary patterns, levels of food safety, resources that support PA, access to health services, and people's beliefs, awareness, and behaviors about healthy eating and lifestyle.

We found gender differences in the SES-obesity relationship, with a negative association between education level and obesity in women, but no such relationship was found in men. Research suggests that there is a social stratification of women's body size (56). Body size ideals and perceptions of social pressure to be slim vary by socioeconomic class. Socially advantaged women are more dissatisfied with their bodies than socially disadvantaged women (57). The desire to be slim will eventually be transformed into powerful motivation to keep a fit figure. Besides, well-educated women have an advantage in understanding the health benefits of a healthy weight, a reasonable diet, and adequate PA (58, 59). Additionally, according to Gore et al., girls with lower education levels had the highest levels of depressive symptoms and stress levels (60), which may lead to pro-inflammatory effects or reduce the integrity of the intestinal barrier, in which case obesity may be induced (61). But to men, high SES not only increases the chance of obtaining excess food, but also increases the chance of avoiding manual labor (62), and larger size generally regarded as more likely to have physical strength and superiority, so the community culture of male obesity is relatively tolerant (58).

Based on our result, we found a negative interaction association between PA and low education level on obesity measures in women, which is consistent with some existing literature (21, 22). For instance, a study conducted in Spain used path analysis to disentangle the direct and indirect effects of SES on obesity, in which PA served as a mediator variable, and found that PA had a significant mediating effect on the SES-obesity relationship and concluded that promoting PA was helpful to prevent obesity (21). The possible mechanisms underlying the combined association of SES indicators and PA on obesity measures deserve exploration. There are several potential explanations. The socio-economic and physical environment could affect people's health both tangibly and intangibly (63, 64), as Adler found that social inequalities such as unequal income distribution increase people's exposure to stressful events (63). Physical environment characteristics, on the other hand, could affect people's opportunities to engage in PA, specifically the availability of parks and recreation facilities, and a pedestrian environment that promotes PA, such as walking and exercise (64), which is, however, very limited in rural areas. With increased exposure to stress and decreased access to PA, obesity is more likely to occur. Another explanation is that, according to Carroll-Scott et al., a disadvantageous SES is itself a stressful state (65), and PA can alleviate oxidative stress or inflammatory response (40, 41, 61), thus to a certain extent offset the adverse effects of SES on obesity.

Our results also found gender differences in the combined association of PA and SES indicators on obesity measures. The possible explanations are as follows. First, several studies have found that low SES has a greater negative impact on good health for women than for men (66). Our results are in line with this finding, with men being largely unaffected in terms of the burden of obesity at low education level, while women show a significant inverse relationship. Furthermore, men with low SES were more likely to engage in high levels of PA (62), making it less likely that low SES and PA would have a meaningful interaction effect on obesity. Secondly, women are less educated than men, with 51.3% of women vs. 33.6% of men classified in the low education group as shown in Table 1. Less-educated women are less likely to work, and roles such as childrearing and maintaining the household are usually fulfilled by them (66), thus they are more likely to be socially isolated compared to men (67). However, mutual support among socially connected people is of great significance as it can lead to positive changes such as reduced caloric intake and increased PA (68). As educational attainment has been established, PA can be an alternative way to reduce the obesity associated with low levels of education, especially among women.

The present study has several strengths: the exploration of the SES-PA-obesity relationship was conducted in a relatively large rural population, which helps to fill a gap in the literature on the SES-PA-obesity relationship in rural areas. Besides, adjusting for multiple covariates helps to control for potential confounders, and defining obesity using several different objectively measured anthropometric measures contributes to more accurate monitoring of obesity. In terms of limitations, first, due to the nature of the cross-sectional design, the present study was unable to determine a causal relationship between SES or PA and obesity measurements. Second, the information on lifestyle factors was obtained based on self-reports, so recall bias cannot be excluded. Finally, although some important confounding covariates were controlled for, some unmeasured factors (e.g., genetic and physiological factors) were not taken into account, which may have influenced the results.

## Conclusion

In summary, the burden of obesity is likely to be higher among women with low levels of education and men with high per capita monthly income in the Chinese rural population. Promoting PA may counteract the negative impact of low SES on obesity in women. From a policy perspective, gender differences need to be considered when taking measures to reduce the prevalence of obesity and reduce the SES gradient, and further prospective studies through geographically robust study designs are needed.

## Data Availability Statement

The raw data supporting the conclusions of this article will be made available by the authors, without undue reservation.

## Ethics Statement

The studies involving human participants were reviewed and approved by Ethics approval was obtained from the Zhengzhou University Life Science Ethics Committee [Ethics approval code: [2015] MEC (S128)]. The patients/participants provided their written informed consent to participate in this study.

## Author Contributions

MP: investigation, data curation, methodology, formal analysis, visualization, and writing-original draft. JH: investigation, data curation, writing-review, and editing. JG, RL, ZM, and WH: investigation, writing-review, and editing. XL: investigation, validation, writing-review, and editing. RC: investigation, methodology, writing-review, and editing. SY and XW: writing-review and editing. XW: data curation, writing-review, and editing. JH: conceptualization, methodology, investigation, validation, supervision, writing-reviewing, and editing. CW: conceptualization, methodology, investigation, validation, supervision, funding acquisition, project administration, and writing-original draft. All authors contributed to the article and approved the submitted version.

## Conflict of Interest

The authors declare that the research was conducted in the absence of any commercial or financial relationships that could be construed as a potential conflict of interest.

## Abbreviations

BFP: body fat percentage
BMI: body mass index
CI: confidence interval
MET: metabolic equivalent
PA: physical activity
SD: standard deviation
SES: socioeconomic status
VFI: visceral fat index
WC: waist circumference
WHR: waist-to-hip ratio
WHtR: waist-to-height ratio.
