# Supplementary material for: Associations of Socioeconomic Status and Physical Activity With Obesity Measures in Rural Chinese Adults
Source: Front Public Health. 2021 Jan 8;8:594874. doi: 10.3389/fpubh.2020.594874 (PMC7820760; doi:10.3389/fpubh.2020.594874)
Supplement: Supplementary file 1 [file Data_Sheet_1.docx]

**
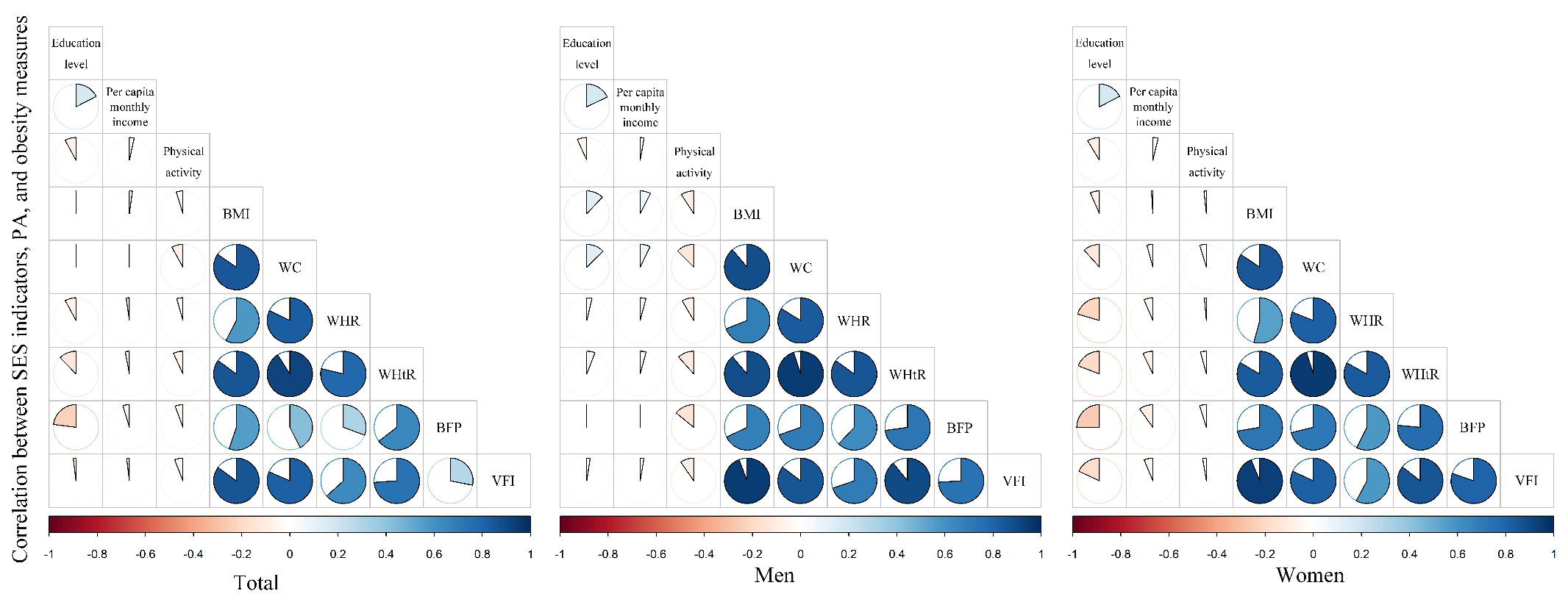
**

**Figure S1 Correlation between SES indicators, PA, and obesity measures.** The correlation maps showed the Pearson Corr. values for each comparison. The bars at the bottom of the maps exhibited the color legend of the Pearson Corr. values for each indicator in the matrix, and the size of the sector area indicated the size of the corresponding correlation coefficient, with different colors representing different correlation directions. Abbreviation: BMI, body mass index; WC, waist circumference; WHR, waist-to-hip ratio; WHtR, waist-to-height ratio; BFP, body fat percentage; VFI, visceral fat index.

Table S1 Associations between SES indicators or PA and obesity measures

|  |  | BMI | | WC | | WHR | | WHtR | | BFP | | VFI | |
| --- | --- | --- | --- | --- | --- | --- | --- | --- | --- | --- | --- | --- | --- |
|  |  | OR (95%) | *P* | OR (95%) | *P* | OR (95%) | *P* | OR (95%) | *P* | OR (95%) | *P* | OR (95%) | *P* |
| Total | Education level |  |  |  |  |  |  |  |  |  |  |  |  |
|  | High | Ref |  | Ref |  | Ref |  | Ref |  | Ref |  | Ref |  |
|  | Low | 1.466(1.337,1.608) | <0.001 | 1.29(1.198,1.389) | <0.001 | 1.383(1.285,1.488) | <0.001 | 1.415(1.312,1.527) | <0.001 | 1.225(1.130,1.327) | <0.001 | 1.238(1.147,1.335) | <0.001 |
|  | Medium | 1.136(1.044,1.237) | 0.003 | 1.141(1.066,1.221) | <0.001 | 1.191(1.114,1.273) | <0.001 | 1.200(1.120,1.286) | <0.001 | 1.029(0.957,1.107) | 0.435 | 1.009(0.941,1.082) | 0.804 |
|  | Per capita monthly income | 1.089(1.015,1.170) | 0.018 | 1.153(1.090,1.220) | <0.001 | 1.113(1.052,1.177) | <0.001 | 1.118(1.055,1.186) | <0.001 | 1.163(1.091,1.240) | <0.001 | 1.097(1.036,1.162) | 0.002 |
|  | Physical activity |  |  |  |  |  |  |  |  |  |  |  |  |
|  | Low | Ref |  | Ref |  | Ref |  | Ref |  | Ref |  | Ref |  |
|  | Moderate | 0.855(0.801,0.913) | <0.001 | 0.898(0.851,0.948) | <0.001 | 0.969(0.918,1.023) | 0.262 | 0.963(0.910,1.019) | 0.197 | 0.861(0.810,0.915) | <0.001 | 0.892(0.844,0.942) | <0.001 |
|  | High | 0.737(0.687,0.792) | <0.001 | 0.774(0.731,0.819) | <0.001 | 0.835(0.789,0.884) | <0.001 | 0.823(0.776,0.874) | <0.001 | 0.658(0.618,0.701) | <0.001 | 0.759(0.716,0.804) | <0.001 |
| Men | Education level |  |  |  |  |  |  |  |  |  |  |  |  |
|  | High | Ref |  | Ref |  | Ref |  | Ref |  | Ref |  | Ref |  |
|  | Low | 1.064(0.924,1.225) | 0.388 | 0.917(0.821,1.024) | 0.126 | 0.970(0.873,1.078) | 0.572 | 0.966(0.867,1.078) | 0.538 | 0.879(0.790,0.978) | 0.018 | 0.866(0.775,0.967) | 0.011 |
|  | Medium | 0.943(0.838,1.062) | 0.332 | 0.979(0.889,1.077) | 0.659 | 1.066(0.971,1.170) | 0.181 | 1.017(0.923,1.121) | 0.732 | 0.848(0.772,0.932) | 0.001 | 0.889(0.805,0.981) | 0.020 |
|  | Per capita monthly income | 1.192(1.055,1.347) | 0.005 | 1.331(1.212,1.462) | <0.001 | 1.256(1.152,1.371) | <0.001 | 1.262(1.154,1.380) | <0.001 | 1.267(1.160,1.385) | <0.001 | 1.228(1.122,1.344) | <0.001 |
|  | Physical activity |  |  |  |  |  |  |  |  |  |  |  |  |
|  | Low | Ref |  | Ref |  | Ref |  | Ref |  | Ref |  | Ref |  |
|  | Moderate | 0.802(0.716,0.899) | <0.001 | 0.868(0.794,0.950) | 0.002 | 0.888(0.815,0.968) | 0.007 | 0.926(0.847,1.012) | 0.091 | 0.842(0.773,0.918) | <0.001 | 0.913(0.835,0.999) | 0.047 |
|  | High | 0.617(0.552,0.689) | <0.001 | 0.665(0.610,0.725) | <0.001 | 0.725(0.668,0.786) | <0.001 | 0.724(0.665,0.787) | <0.001 | 0.590(0.543,0.640) | <0.001 | 0.737(0.677,0.801) | <0.001 |
| Women | Education level |  |  |  |  |  |  |  |  |  |  |  |  |
|  | High | Ref |  | Ref |  | Ref |  | Ref |  | Ref |  | Ref |  |
|  | Low | 1.853(1.625,2.114) | <0.001 | 1.606(1.452,1.776) | <0.001 | 1.774(1.601,1.966) | <0.001 | 1.878(1.689,2.089) | <0.001 | 1.705(1.511,1.923) | <0.001 | 1.648(1.474,1.843) | <0.001 |
|  | Medium | 1.459(1.287,1.654) | <0.001 | 1.397(1.271,1.536) | <0.001 | 1.415(1.285,1.557) | <0.001 | 1.486(1.346,1.639) | <0.001 | 1.370(1.229,1.527) | <0.001 | 1.236(1.109,1.377) | <0.001 |
|  | Per capita monthly income | 1.038(0.951,1.133) | 0.406 | 1.050(0.976,1.1290) | 0.191 | 1.013(0.939,1.093) | 0.740 | 1.012(0.934,1.096) | 0.777 | 1.043(0.948,1.148) | 0.385 | 1.021(0.948,1.100) | 0.586 |
|  | Physical activity |  |  |  |  |  |  |  |  |  |  |  |  |
|  | Low | Ref |  | Ref |  | Ref |  | Ref |  | Ref |  | Ref |  |
|  | Moderate | 0.891(0.822,0.967) | 0.006 | 0.911(0.850,0.976) | 0.008 | 1.019(0.949,1.095) | 0.602 | 0.988(0.916,1.065) | 0.751 | 0.880(0.805,0.961) | 0.005 | 0.886(0.826,0.951) | 0.001 |
|  | High | 0.823(0.750,0.903) | <0.001 | 0.853(0.789,0.922) | <0.001 | 0.938(0.864,1.017) | 0.120 | 0.915(0.840,0.996) | 0.041 | 0.743(0.672,0.822) | <0.001 | 0.772(0.713,0.837) | <0.001 |
| Abbreviation: SES, socioeconomic status; PA, physical activity; BMI, body mass index; WC, waist circumference; WHR, waist-to-hip ratio; WHtR, waist-to-height ratio; BFP, body fat percentage; VFI, visceral fat index. Logistic regression models were used to assess the associations of SES indicators or PA with obesity (OR, 95%CI) in a fully adjusted model, which adjusted for region, age, marital status, smoking status, drinking status, fruit and vegetable intake, high fat diet, SES indicators or PA. | | | | | | | | | | | | | |

**Table S2 Associations between SES indicators or PA and obesity defined by different cut-off values of BMI in sensitivity analysis.**

|  |  | Obesity (BMI ≥28kg/m^2^) | |  | Obesity (BMI ≥30kg/m^2^) | |
| --- | --- | --- | --- | --- | --- | --- |
|  |  | OR (95%CI) | *P* |  | OR (95%CI) | *P* |
| Total | Education level |  |  |  |  |  |
|  | High | Ref.. |  |  | Ref. |  |
|  | Medium | 1.136(1.044,1.237) | 0.003 |  | 1.245(1.100,1.408) | 0.001 |
|  | Low | 1.466(1.337,1.608) | <0.001 |  | 1.744(1.525,1.994) | <0.001 |
|  | Per capita monthly income | 1.089(1.015,1.170) | 0.018 |  | 1.052(0.951,1.164) | 0.325 |
|  | Physical activity |  |  |  |  |  |
|  | Low | Ref. |  |  | Ref. |  |
|  | Moderate | 0.855(0.801,0.913) | <0.001 |  | 0.829(0.755,0.910) | <0.001 |
|  | High | 0.737(0.687,0.792) | <0.001 |  | 0.719(0.650,0.796) | <0.001 |
| Men | Education level |  |  |  |  |  |
|  | High | Ref. |  |  | Ref. |  |
|  | Medium | 0.943(0.838,1.062) | 0.332 |  | 0.967(0.815,1.149) | 0.704 |
|  | Low | 1.064(0.924,1.225) | 0.388 |  | 1.123(0.913,1.382) | 0.273 |
|  | Per capita monthly income | 1.192(1.055,1.347) | 0.005 |  | 1.140(0.951,1.366) | 0.157 |
|  | Physical activity |  |  |  |  |  |
|  | Low | Ref. |  |  | Ref. |  |
|  | Moderate | 0.802(0.716,0.899) | <0.001 |  | 0.761(0.644,0.900) | 0.001 |
|  | High | 0.617(0.552,0.689) | <0.001 |  | 0.612(0.520,0.720) | <0.001 |
| Women | Education level |  |  |  |  |  |
|  | High | Ref. |  |  | Ref. |  |
|  | Medium | 1.459(1.287,1.654) | <0.001 |  | 1.688(1.403,2.031) | <0.001 |
|  | Low | 1.853(1.625,2.114) | <0.001 |  | 2.382(1.962,2.891) | <0.001 |
|  | Per capita monthly income | 1.038(0.951,1.133) | 0.406 |  | 1.011(0.895,1.142) | 0.856 |
|  | Physical activity |  |  |  |  |  |
|  | Low | Ref. |  |  | Ref. |  |
|  | Moderate | 0.891(0.822,0.967) | 0.006 |  | 0.862(0.77,0.965) | 0.010 |
|  | High | 0.823(0.750,0.903) | <0.001 |  | 0.782(0.687,0.89) | <0.001 |
| Abbreviation: SES, socioeconomic status; PA, physical activity; BMI, body mass index; WC, waist circumference; WHR, waist-to-hip ratio; WHtR, waist-to-height ratio; BFP, body fat percentage; VFI, visceral fat index. Logistic regression analyses were performed using a fully adjusted model that adjusted for region, age, marital status, smoking status, drinking status, fruit and vegetable intake, high fat diet, SES indicators or PA. | | | | | | |
